# Sex- and reward-dependent effects of early life scarcity-adversity on adolescent behavioral responses to natural rewards

**DOI:** 10.3389/fnbeh.2025.1659339

**Published:** 2025-09-01

**Authors:** Melissa G. Salazar, Christine H. Nguyen, Sasha Oak, Jeffy Jackson, Millie Rincón-Cortés

**Affiliations:** Department of Neuroscience, School of Behavioral and Brain Sciences, University of Texas at Dallas, Richardson, TX, United States

**Keywords:** early life adversity, development, reward, sucrose, palatable food, play, adolescence, sex differences

## Abstract

Early life adversity (ELA) increases risk for multiple psychiatric disorders that are characterized by reward-related dysfunction. Disrupted reward-related processes are commonly observed in humans and rodents following ELA. Rodent studies have shown sex differences in response to natural and drug rewards at baseline, following ELA, and in rodent models of psychiatric diseases that are potentiated by ELA. Yet, less is known regarding the development of ELA-induced alterations in reward-related responses, including how these may differ by sex. To this end, we tested behavioral responses to consummatory and social rewards in control and scarcity-adversity male and female rats using sucrose preference, palatable food consumption, and social play tests during peripuberty and adolescence. Our results suggest no impact of early life scarcity-adversity during peripuberty, but sex- and reward-dependent adolescent effects in which females display reduced sucrose preference whereas males display lower levels of social play solicitations (i.e., dorsal contacts). These findings suggest age-, sex-, and reward-specific effects of early life scarcity-adversity in response to consummatory and social rewards, which appear to emerge during adolescence.

## 1 Introduction

Early life experiences fundamentally shape the trajectory of behavioral and brain development throughout the lifespan (Mcewen, 2003; Berens et al., 2017; Nelson and Gabard-Durnam, 2020). Among the most influential factors during this critical period is the quality of maternal care, which serves as a primary mediator between environmental conditions and offspring development (Hofer, 1978; Levine, 2001; Rincon-Cortes and Sullivan, 2014; Curley and Champagne, 2016). The early postpartum period in rodents, typically spanning the first week to 10 days after giving birth, represents a critical window during which environmental adversity can fundamentally alter maternal caregiving patterns (Ivy et al., 2008; Rincon-Cortes and Grace, 2022). In accordance, resource scarcity paradigms that limit access to nesting materials during this period consistently produce alterations in maternal behavior characterized by increased fragmentation of maternal care and elevated rates of adverse pup-directed behaviors (Ivy et al., 2008; Gallo et al., 2019; Rincon-Cortes and Grace, 2022; Porras et al., 2025).

Importantly, environmentally induced alterations in maternal behavior create cascading and enduring effects in the offspring (Walker et al., 2017; Glynn and Baram, 2019; Rincon-Cortes, 2025). For example, studies utilizing resource scarcity paradigms have consistently demonstrated that offspring exposed to limited nesting material conditions during early postnatal life exhibit profound alterations in reward-seeking and consummatory behaviors that persist across development and differ by sex (Birnie et al., 2022; Duque-Quintero et al., 2022; Williams et al., 2022; Rincon-Cortes, 2023). Indeed, early life adversity (ELA) in the form of resource scarcity (and the resulting disruption in mother-infant interactions) typically reduces sucrose preference/consumption in adult male rats, but not in female rats, and reduces palatable food consumption in males, but increases palatable food consumption in females, demonstrating bidirectional effects (Machado et al., 2013; Molet et al., 2016; Bolton et al., 2018a; Bolton et al., 2018b; Levis et al., 2021; Kangas et al., 2022). Additionally, this form of ELA decreases social play behaviors in male rats, although effects on females remain unknown (Molet et al., 2016; Bolton et al., 2018a). However, most existing studies have focused exclusively on adult outcomes, potentially obscuring important sex-specific effects that emerge earlier in development. Furthermore, the temporal dynamics of how ELA influences responses to different types of natural rewards—consummatory versus social rewards—across development remain poorly understood.

The focus on adult outcomes in previous studies represents a significant limitation considering that the brain’s reward system undergoes extensive development during adolescence, with continued maturation extending well into early adulthood (Galvan, 2010; Sturman and Moghaddam, 2011; Doremus-Fitzwater and Spear, 2016; Reynolds and Flores, 2021). This protracted developmental timeline creates windows of vulnerability during which ELA can exert lasting influences on reward-related behavioral and brain function (Rodrigues et al., 2011; Birnie et al., 2020; Hanson et al., 2021; Duque-Quintero et al., 2022). Both consummatory rewards, such as sugar-containing foods and liquids, and social rewards, including play behavior, engage overlapping but distinct behavioral systems that are sensitive to early life perturbations (Kelley and Berridge, 2002; Birnie et al., 2022). Emerging evidence suggests that the effects of ELA on reward-related behaviors may manifest in a sex-specific manner (Birnie et al., 2022; Duque-Quintero et al., 2022; Williams et al., 2022; Rincon-Cortes, 2023), but few investigations have systematically examined these effects during the critical developmental periods of peripuberty and adolescence. Thus, the specific developmental trajectories of reward-related behaviors following ELA remain incompletely characterized, particularly with regards to potential sex differences. This knowledge gap is significant given that adolescence represents a period of heightened reward sensitivity and increased vulnerability to the development of addictive behaviors, particularly after ELA exposure (Andersen and Teicher, 2009; Doremus-Fitzwater et al., 2010; Levis et al., 2022).

The present study addresses these critical gaps by systematically examining the effects of early life scarcity-adversity on reward-related behaviors in rats of both sexes across two key developmental periods: peripuberty and adolescence. Briefly, we employed a well-characterized resource scarcity paradigm during the early postpartum period, which results in aberrant pup-directed maternal behavior (Rincon-Cortes and Grace, 2022; Lauraine et al., 2024; Porras et al., 2025), and conducted comprehensive behavioral assessments of both consummatory and social reward in developing male and female offspring (Oak et al., 2024). This approach enables identification of sex-specific and reward-type-specific effects that may emerge at different developmental timepoints. Given the established literature regarding ELA effects on adult reward processing (Birnie et al., 2022; Duque-Quintero et al., 2022; Williams et al., 2022; Rincon-Cortes, 2023), we hypothesized that early life scarcity-adversity would produce sex-dependent alterations in reward-related behaviors, with effects potentially differing between peripuberty and adolescence.

## 2 Materials and methods

### 2.1 Animals

Adult (250–300 g) primiparous female Sprague–Dawley rats were bred in house and maintained in a temperature-controlled room on a 12-h light/dark cycle (6:00 AM lights on/6:00 PM lights off) with access to food and water *ad libitum*. Breeding entailed cohousing a female with an adult male breeder for a 3-week period after which the pregnant female was separated and single-housed. Parturition was verified daily (2-3x per day) during the light cycle from gestational days 20–23. The day of birth was designated as postnatal day (PND) 0 and litters were culled to 10 pups with equal distribution (5 males and 5 females) or roughly equal sex distribution (6 males, 4 females) on PND 2. Litters underwent control (CON) or scarcity-adversity (ELA) conditions from PND 2–9. Both male and female rats were weaned on PND 23, which is consistent with our previous studies (Rincon-Cortes and Grace, 2022; Oak et al., 2024; Nguyen et al., 2025). Weaned rats were then pair-housed with same-sex littermates of the same condition and transferred to a reverse light/dark cycle (6 AM lights off/6 PM lights on) where they remained until the end of behavioral testing. To control for litter effects, only one animal of each sex from a given litter was used for behavioral testing at each of the time points (i.e., peripuberty, adolescence). 9–10 litters were used per condition. A total of 95 animals (9 control dams, 10 scarcity-adversity dams, 36 control offspring, 40 ELA offspring) were used for these experiments. All experiments were carried out according to NIH guidelines and were approved by the University of Texas at Dallas Institutional Animal Care and Use Committee.

### 2.2 Scarcity-adversity paradigm and maternal behavior observations

The early life scarcity-adversity paradigm consisted of reducing the amount of bedding and nesting materials available in the home cage from PND 2–9 (Rincon-Cortes and Grace, 2022; Lauraine et al., 2024; Nguyen et al., 2025; Porras et al., 2025). Litters assigned to the scarcity-adversity condition received 500 mL of corncob bedding and no nestlet material, while litters assigned to the control condition received 1800 mL of corncob bedding and a nestlet. This scarcity-adversity paradigm results in an impoverished nesting environment for the rat dam that provokes changes in pup-directed maternal behaviors, thereby resulting in a form of ELA for the pups (Walker et al., 2017; Rincon-Cortes and Grace, 2022; Rincon-Cortes, 2024, 2025).

Maternal behaviors were observed for 30 min in the home cage twice a day (morning session, afternoon session) from PND 2–5. PND 2–5 was selected for maternal observations based on prior studies finding the most robust behavioral effects occurring during this period (Rincon-Cortes and Grace, 2022; Lauraine et al., 2024). Maternal behaviors recorded included: time spent in nest, nursing, licking, anogenital licking, nest-building, stepping on pups, dragging pups (i.e., pups nipple attached while dam moves around the cage), shoving pups (i.e., dam using head or paws to push pups away), transporting pups (i.e., dam picks pup up with their mouth and relocates them away/out of nest), and biting/chasing tail- a stress-related behavior in rodents (Kubo et al., 2015). Dams were spot-checked for each behavior multiple times per minute within each 5-min segment of each 30-min observation period. If a behavior was observed, a tally mark was made on that segment. Percentages of behaviors were calculated by dividing the number of segments in which a particular behavior occurred over the total number of observation segments on all days by an experimenter blinded to the dam’s experimental condition (Rincon-Cortes and Grace, 2022; Lauraine et al., 2024; Nguyen et al., 2025; Porras et al., 2025).

### 2.3 Behavioral testing

**Figure 1:**
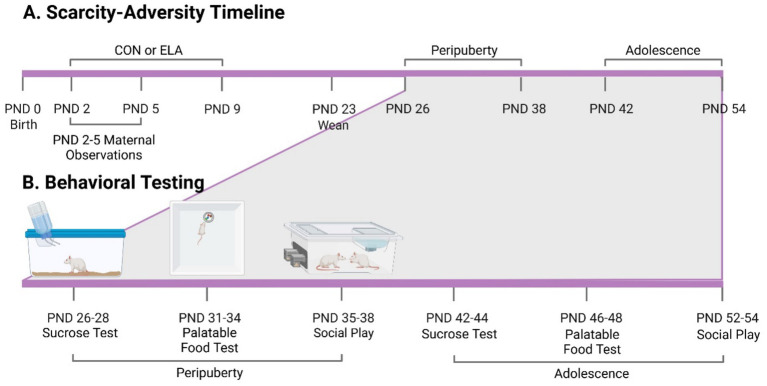
Experimental timeline and design. **(A)** Day of birth was assigned as postnatal day (PND) 0. Following birth, litters were exposed to either the control (CON) or scarcity-adversity (ELA) condition from PND 2–9. Four offspring per sex (2 pairs of males, 2 pairs of females) per litter were weaned and kept for behavioral testing during peripuberty or adolescence. **(B)** Depiction of behavioral testing timeline. Two of the male and female offspring (one male and one female per timepoint) were used for behavioral testing during peripuberty or adolescence, but not both. All rats were tested for behavior in the same order: sucrose preference test, palatable food test, and social play. Figure created with BioRender.com.

The behavioral testing procedure was adapted from a previously published study examining behavioral responses natural prior to rewards in male and female animals during peripuberty and adolescence (Oak et al., 2024). Male and female rats underwent a behavioral test battery for natural rewards during peripuberty (PND 26–38) or adolescence (PND 42–54) (see Figure 1, Experimental Timeline and Design). In this study, peripuberty refers to the period of time from PND 28–38, which is thought to cover the equivalent of “childhood” (PND28-30) and around puberty onset (PND34-P42) in the rat (Rivest, 1991; Marquez et al., 2013; Tzanoulinou et al., 2014a; Tzanoulinou et al., 2014b; Fuochi et al., 2022). This peripubertal period corresponds to childhood in humans (Quinn, 2005; Sengupta, 2013). We use adolescence to refer to animals that are post-pubertal but have not reached adulthood (>P60) (McCutcheon and Marinelli, 2009), corresponding to early-to-mid human adolescence (Quinn, 2005). Different animals from the same litter were used for each of the two timepoints so that each animal was only tested at one timepoint (either peripuberty or adolescence). All behavioral testing occurred between 12:00 PM and 4:00 PM in the home cage to minimize novelty and stress exposure. All behavioral tests occurred during the animal’s dark cycle under red light. All animals received behavioral testing in the following order: sucrose preference, palatable food consumption, and social play.

#### 2.3.1 Sucrose preference

Rats of both sexes were tested during peripuberty (PND 26–28) or adolescence (PND 42–44). Prior to testing, rats were given a 24-h habituation period during which they were presented with two bottles in the home cage: one containing water and the other containing a 1% sucrose solution in order to prevent neophobia during testing (Corey, 1978). Bottle positions were switched twice (every 4–5 h prior to 6:00 PM) during habituation to prevent the formation of location preference. A 1% concentration of sucrose was selected based on previous studies examining ELA-induced changes in sucrose preference and/or consumption during adolescence (Molet et al., 2016; Doherty et al., 2017; Yan et al., 2017). On test day, rats were single-housed and underwent a 3-h food and water deprivation immediately prior to a 1-h test in the home cage. Sucrose preference was calculated based on changes in bottle weight using the following formula: sucrose intake (g)/total sucrose + water intake (g) x 100%.

#### 2.3.2 Palatable food consumption

Rats of both sexes were tested during peripuberty (PND 31–34) or adolescence (PND 46–48). Rats underwent a ~ 3.5-h habituation to chocolate candy pieces (M&Ms), which were placed inside a ramekin in the home cage the day before testing. This was done to avoid confounds of novelty on feeding behavior and hedonic drive, as both male and female rats consume less of a novel food in a familiar environment and consume less food in a novel environment (Greiner and Petrovich, 2020). On the day after the habituation, animals were single-housed and underwent a 3-h food deprivation immediately prior to a 1-h test in the home cage in which rats were tested individually. Chocolate candy intake was determined by calculating the difference in pre- and post-test candy weight (Bolton et al., 2018b; Oak et al., 2024).

#### 2.3.3 Social play

Rats of both sexes were tested during peripuberty (PND 35–38) or adolescence (PND 52–54). On test day, rats were separated from their cage mate (matched for age, sex, and condition) for a 3-h isolation period, which was done to increase motivation for social play to half maximal levels and prevent floor and ceiling effects (Niesink and Van Ree, 1989; Vanderschuren et al., 1995). During this time, each animal was placed into a clean cage with ample food and water. Following the 3-h isolation period, animals were reunited with their cage mate by placing both animals into their home cage (in the absence of food and water) (Oak et al., 2024). Behavior was recorded immediately after reuniting the animals over a 10-min period using a video camera. The frequency of dorsal contacts (also known as nape attacks or pouncing) and the number of pins were scored as a pair by an experimenter that was blinded to the animal’s condition. Dorsal contacts, which are considered an index of play solicitations, were scored when one rat contacted the other rat’s nape or the upper half of the body either with its paw, two paws, or snout/mouth (Meaney and Stewart, 1981). Pinning was scored when one rat was lying on its back or side (with most of its back to the ground), while the other rat stood over it (Panksepp and Beatty, 1980; Panksepp, 1981). These specific behaviors (e.g., dorsal contacts, pins) are considered the main indices of social play behaviors (Panksepp and Beatty, 1980; Vanderschuren and Trezza, 2014).

### 2.4 Statistical analysis

Data sets with a normal distribution were analyzed using unpaired *t*-tests; data sets deviating from the normal distribution were analyzed using Mann–Whitney *U* tests. For all analyses, we compared control males versus ELA males and control females versus ELA females separately to examine sex-specific ELA effects. We did not include direct between-sex comparisons (males versus females within treatment groups) as our previous study found no basal sex differences between control males and control females at the timepoints used in this study (Oak et al., 2024). Statistics were calculated using GraphPad Prism 10 and differences were considered significant when *p* < 0.05. Statistical outliers were identified using Grubbs test and excluded from analysis. Sample sizes were determined *a priori* by conducting a power analysis using means and standard deviations (and an 80% power threshold) from preliminary data showing reduced dorsal contacts in ELA animals. The means for dorsal contacts in CON and ELA rats were 32.50 and 20.00 and the standard deviations were 9.27 and 9.35, respectively. Using G*Power (Faul et al., 2007; Kang, 2021), we determined that we needed an *n* = 10 rats per group to detect a significant difference (*p* < 0.05) at ≥ 80% power.

## 3 Results

### 3.1 Postpartum resource scarcity increases adverse pup-directed maternal behaviors

To confirm that postpartum scarcity-adversity produced changes in pup-directed maternal behavior, we conducted home cage maternal behavior observations (Table 1). Compared to control dams (*n* = 8–9), scarcity-adversity dams (*n* = 9–10) exhibited an increase in the percentage of time spent in the nest (t_17_ = 3.16, *p* = 0.006). However, control and scarcity-adversity dams exhibited comparable percentages of time spent nursing (t_17_ = 1.94, *p* = 0.07) and licking pups (t_17_ = 1.07, *p* = 0.30). Compared to control dams, scarcity-adversity dams exhibited an increase in the percentage of time spent on anogenital licking of pups (t_17_ = 2.15, *p* = 0.04). Control and scarcity-adversity dams exhibited comparable percentages of time spent nest building (t_17_ = 0.81, *p* = 0.43). However, scarcity-adversity dams showed an increase in percentage of time spent stepping on pups (*U* = 0, *p* < 0.0001), dragging pups (*U* = 9, *p* = 0.002), and shoving pups (*U* = 7, *p* = 0.0008), but no differences in time spent transporting pups (*U* = 22, *p* = 0.06). Moreover, scarcity-adversity dams spent more time biting or chasing their tail (U = 0, *p* < 0.0001).

**Table 1:** Summary of statistics for maternal behavior observations.

Maternal behaviors observed in CON and ELA Litters (%)			
Behavior	CON	ADV	*p*-value
Time Spent in Nest	47.45 ± 14.43	68.12 ± 14.10**	*p* = 0.006
Nursing Pups	34.26 ± 14.48	46.25 ± 12.53	*p* = 0.07
Licking Pups	21.89 ± 11.20	27.50 ± 11.53	*p* = 0.30
Anogenital Licking	19.44 ± 9.54	26.87 ± 5.05*	*p* = 0.04
Nest Building	10.95 ± 5.09	14.18 ± 10.92	*p* = 0.43
Stepping on Pups	1.04 ± 1.11	16.25 ± 10.24****	*p* < 0.0001
Dragging Pups	5.32 ± 5.62	17.29 ± 8.10***	*p* = 0.0002
Shoving Pups	1.39 ± 1.80	10.00 ± 6.86***	*p* = 0.0008
Transporting Pups	2.08 ± 3.61	6.46 ± 5.51	*p* = 0.06
Chasing/Biting Tail	0.00 ± 0.00	7.18 ± 5.81****	*p* < 0.0001

Mean ± standard deviation (SD) is shown for numerical variables. *p < 0.05, **p < 0.01, ***p < 0.001, ****p < 0.0001.

### 3.2 No impact of early life scarcity-adversity on behavioral responses to consummatory or social rewards during peripuberty

**Figure 2:**
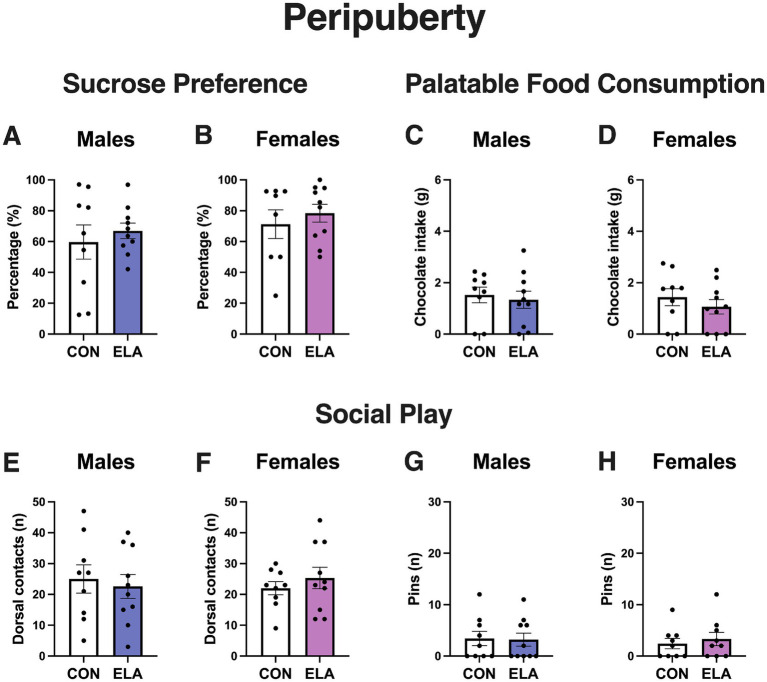
Early life scarcity-adversity produces similar behavioral responses to consummatory and social rewards in peripubertal male and female rats. **(A)** Peripubertal control (CON) and scarcity-adversity (ELA) male rats exhibited similar levels of sucrose preference (*p* = 0.55). **(B)** Peripubertal CON and ELA female rats exhibited similar levels of sucrose preference (*p* = 0.51). **(C)** Chocolate candy intake was similar in peripubertal CON and ELA male rats (*p* = 0.59). **(D)** Chocolate candy intake was similar in peripubertal CON and ELA female rats (*p* = 0.40). **(E)** Peripubertal CON and ELA male rats displayed comparable levels of play solicitations (*p* = 0.69). **(F)** Peripubertal CON and ELA female rats exhibited comparable levels of play solicitations (*p* = 0.44). **(G)** CON and ELA male rats made similar numbers of pins (*p* = 0.88) during peripuberty. **(H)** CON and ELA female rats made similar numbers of pins (*p* = 0.67) during peripuberty. Error bars represent mean ± SEM. White bars represent control groups, indigo bars represent ELA males, pink bars represent ELA females. *n* = 8–10 rats per group.

Early life scarcity-adversity did not alter behavioral responses to consummatory rewards during peripuberty (Figure 2). In male rats, control (*n* = 9) and scarcity-adversity (*n* = 10) groups showed similar levels of sucrose preference (CON: 59.72% ± 33.29; ELA: 66.95% ± 15.72; t_17_ = 0.61, *p* = 0.55; Figure 2A). In female rats, control (*n* = 8 rats after excluding 1 statistical outlier) and scarcity-adversity (*n* = 10) groups also exhibited comparable levels of sucrose preference (CON: 71.25% ± 26.20; ELA: 78.37% ± 18.29; *U* = 32, *p* = 0.51; Figure 2B). Similarly, no between group differences were found for sweet palatable food consumption in males (CON: 1.52 g ± 0.92, *n* = 9; ELA: 1.34 g ± 1.05, *n* = 10; *U* = 38, *p* = 0.59) or females (CON: 1.44 g ± 1.00, *n* = 9; ELA: 1.06 g ± 0.89, *n* = 10; t_17_ = 0.86, *p* = 0.40; Figures 2C,D).

Early life scarcity-adversity did not result in differences in reward-related social play behaviors during peripuberty in either sex (Figures 2E,H). Control (*n* = 9) and scarcity-adversity (*n* = 10) males displayed comparable levels of dorsal contacts (CON: 25.00 ± 13.65; ELA: 22.60 ± 12.26; t_17_ = 0.40, *p* = 0.69) during peripuberty (Figure 2E). Peripubertal control (*n* = 9) and scarcity-adversity (*n* = 10) female rats also displayed comparable levels of dorsal contacts (CON: 22.00 ± 6.42; ELA: 25.30 ± 11.08; t_17_ = 0.78, *p* = 0.44; Figure 2F). Control (*n* = 9) and scarcity-adversity (*n* = 10) male rats made similar numbers of pins (CON: 3.44 ± 4.22; ELA: 3.20 ± 3.99; *U* = 43, *p* = 0.88; Figure 2G). In females, control (*n* = 9) and scarcity-adversity (n = 9 rats after excluding 1 statistical outlier) peripubertal rats displayed comparable levels of pins (CON: 2.44 ± 3.00; ELA: 3.33 ± 3.90; *U* = 35.50, *p* = 0.67; Figure 2H).

### 3.3 Sex-dependent impact of early life scarcity-adversity on behavioral responses to consummatory and social rewards during adolescence

**Figure 3:**
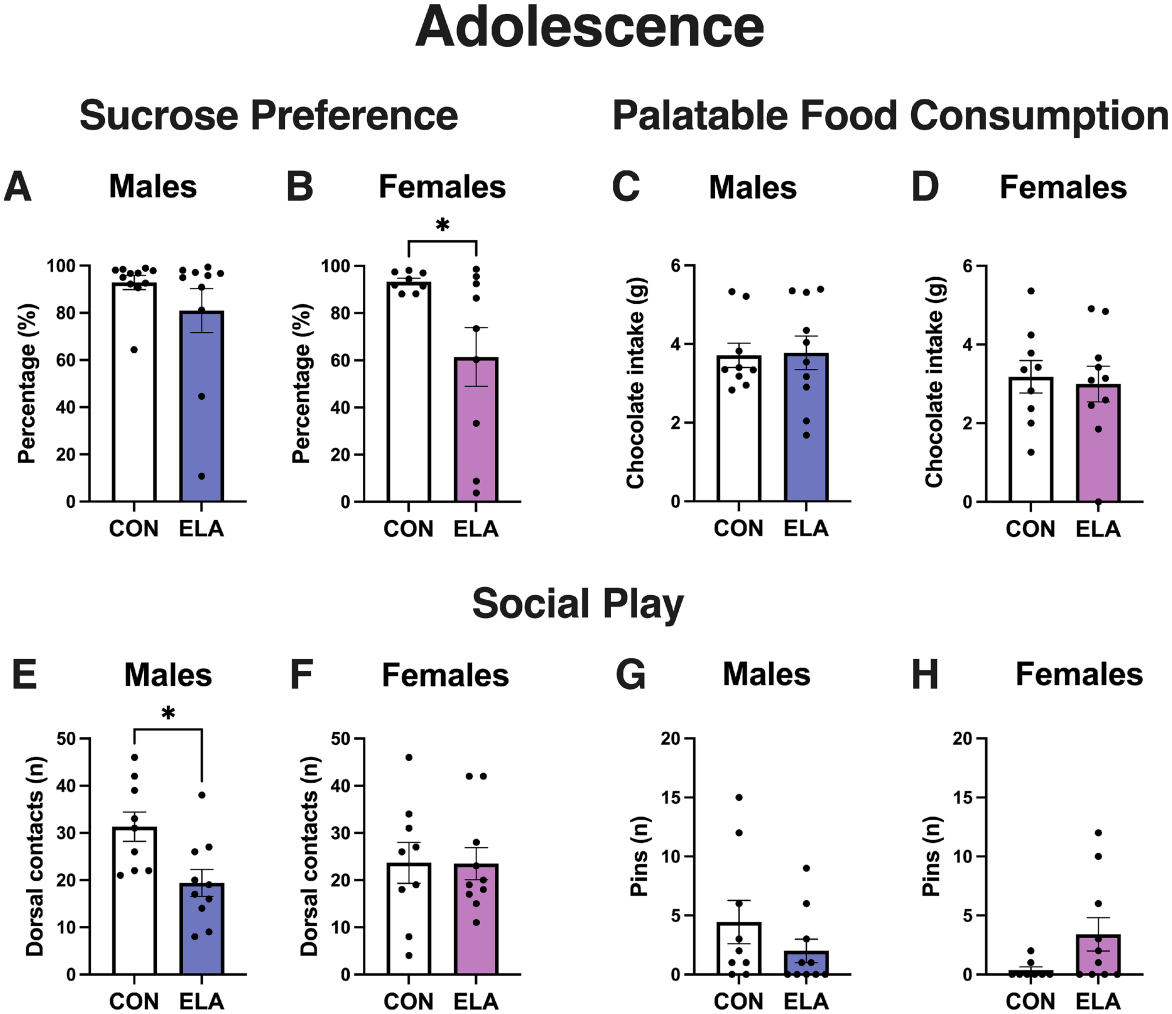
Sex-specific impact of early life scarcity-adversity on consummatory and social rewards during adolescence. **(A)** Adolescent CON and ELA male exhibited similar levels of sucrose preference (*p* = 0.45). **(B)** Compared to adolescent CON rats, ELA female rats exhibited decreased sucrose preference (*p* = 0.03). **(C)** Chocolate candy intake was similar in adolescent CON and ELA male rats (*p* = 0.78). **(D)** Chocolate candy intake was similar in adolescent CON and ELA female rats (*p* = 0.77). **(E)** Compared to CON male rats, ELA male rats displayed lower levels of play solicitations (i.e., dorsal contacts) during adolescence (*p* = 0.01). **(F)** CON and ELA female rats displayed comparable levels of dorsal contacts during adolescence (*p* = 0.83). **(G)** Adolescent CON and ELA male rats made similar numbers of pins (*p* = 0.21) during social play. **(H)** Adolescent CON and ELA female rats made similar numbers of pins (*p* = 0.09) during social play. Error bars represent mean ± SEM. White bars represent control groups, indigo bars represent ELA males, pink bars represent ELA females. *n* = 8–11 rats per group. * *p* < 0.05.

Early life scarcity-adversity decreased reward-related consummatory behaviors in response to sucrose only in adolescent female rats (Figure 3). In males, the control (*n* = 11) and scarcity-adversity (*n* = 10) groups showed similar levels of sucrose preference (CON: 95.74% ± 2.97; ELA: 88.75% ± 17.43; *U* = 35, *p* = 0.45; Figure 3A). In females, the scarcity-adversity (*n* = 9 after excluding 1 animal due to a technical error) group exhibited decreased sucrose preference (CON: 93.35% ± 4.02; ELA: 61.41% ± 37.37; t_15_ = 2.40, *p* = 0.03) compared to controls (*n* = 8 after excluding 1 statistical outlier) (Figure 3B). In males, no differences between control (*n* = 9) and scarcity-adversity groups (*n* = 10) were found for sweet palatable food consumption (CON: 3.71 g ± 0.93; ELA: 3.78 g ± 1.35; U = 41, *p* = 0.78; Figure 3C). Similarly, no differences were found between control (*n* = 9) and scarcity-adversity (n = 10) females for sweet palatable food consumption (CON: 3.18 g ± 1.23; ELA: 2.99 g ± 1.43; t_17_ = 0.30, *p* = 0.77; Figure 3D).

Early life scarcity-adversity had a sex-specific impact on social play in adolescent male rats (Figures 3E–H). Compared to control males (*n* = 9), scarcity-adversity males (*n* = 10) displayed a reduction in the number of dorsal contacts (CON: 31.33 ± 9.35; ELA: 19.40 ± 9.02; t_17_ = 2.83, *p* = 0.01; Figure 3E). In adolescent females, control (*n* = 9) and scarcity-adversity (n = 10) rats displayed comparable numbers of dorsal contacts (CON: 23.67 ± 13.05; ELA: 23.50 ± 10.74; *U* = 42, *p* = 0.83; Figure 3F). In males, control (*n* = 9) and scarcity-adversity (n = 10) rats displayed comparable levels in the number of pins (CON: 4.44 ± 5.50; ELA: 2.00 ± 3.13; *U* = 30, *p* = 0.21; Figure 3G). In females, control (*n* = 8 after excluding 1 statistical outlier) and scarcity-adversity (*n* = 10) rats displayed comparable levels of pins (CON: 0.37 ± 0.74; ELA: 3.40 ± 4.45; *U* = 22, *p* = 0.08; Figure 3H).

## 4 Discussion

Although previous work has demonstrated sex-specific effects of early life resource scarcity and aberrant maternal caregiving on reward-related behaviors during adulthood (Birnie et al., 2022; Williams et al., 2022; Rincon-Cortes, 2023), less is known in developing male and female rats. To this end, we investigated the effects of early life scarcity-adversity on dam-pup interactions and behavioral responses to consummatory and social rewards during peribuberty and adolescence in rats of both sexes. Our findings replicate prior studies indicating aberrant pup-directed maternal behaviors in rat dams exposed to resource scarcity during the early postpartum and extend prior work by showing sex- and reward-specific effects in the adolescent offspring.

### 4.1 Postpartum scarcity-adversity increases adverse pup-directed maternal behaviors

Consistent with prior studies from our group and others, postpartum scarcity-adversity increased adverse pup-directed maternal behaviors (Roth et al., 2009; Raineki et al., 2012; Doherty et al., 2017; Gallo et al., 2019; Perry et al., 2019; Rincon-Cortes and Grace, 2022; Lauraine et al., 2024; Nguyen et al., 2025; Porras et al., 2025). Specifically, scarcity-adversity dams exhibited increased percentages of time spent stepping on, dragging, and shoving pups compared to control dams. Additionally, scarcity-adversity dams exhibited increased tail biting - a form of stress-coping in rodents (Kubo et al., 2015), which is consistent with prior reports (Shupe and Clinton, 2021; Lauraine et al., 2024; Nguyen et al., 2025).

### 4.2 Age- and sex-specific impact of early life scarcity-adversity on consummatory and social rewards

Our findings reveal that early life scarcity-adversity produces distinct, sex-specific alterations in reward-related behaviors that appear to emerge during adolescence. During peripuberty, early life scarcity-adversity had no impact on behavioral responses to consummatory rewards (sucrose solution or chocolate candy) or social rewards (play), as indexed by no between group differences in either sex at this timepoint. This is consistent with a previous study showing no effects of aberrant caregiving/early life resource scarcity on sucrose preference or social approach around PND 30 in either sex (Doherty et al., 2017).

During adolescence, we found a sex-specific reduction in sucrose preference in scarcity-adversity female rats compared to their control counterparts, but no differences between scarcity-adversity and control males. These findings challenge previous research that pooled sexes together, which may have obscured important sex-specific vulnerabilities. For example, a previous study that pooled male and female rats found reduced adolescent sucrose consumption in rats exposed to aberrant caregiving due to early life resource scarcity (Yan et al., 2017). It is possible that the observed effects in this study were driven by females. Interestingly, a previous study in adult female rats found no differences in sucrose consumption and/or preference between female rats exposed to early life resource scarcity and controls (Levis et al., 2021), which may suggest an age-specific effect of early life scarcity-adversity on sucrose preference in female rats. This temporal specificity of the female-specific reduction in sucrose preference during adolescence may suggest that early life scarcity-adversity programs latent changes in reward systems that only manifest during a critical adolescent window when interacting with sex-specific developmental processes (Andersen and Teicher, 2008; Sinclair et al., 2014), potentially involving pubertal hormonal changes or differential maturation of dopaminergic reward systems that normalize by adulthood (Spear, 2000; Wilmouth and Spear, 2009; Friemel et al., 2010).

We found no impact of early life scarcity-adversity on adolescent palatable food consumption. However, previous studies using the limited bedding and nesting (LBN) paradigm, which involves resource scarcity plus the use of an aluminum mesh floor, during early life have found a reduction in palatable food consumption in adult male rats compared to control males (Bolton et al., 2018a), but not in adult females (Levis et al., 2021). The absence of early life scarcity-adversity effects on palatable food consumption during early developmental periods (e.g., peripuberty, adolescence) contrasts with the sex-specific alterations observed in adulthood, suggesting that the impact of early life scarcity-adversity on feeding behavior may emerge through developmental processes that unfold over extended timeframes. It is important to note that test order was not counterbalanced, and sucrose preference testing preceded the palatable food consumption test. Thus, it is possible that prior sucrose exposure may have influenced sensitivity to palatable rewards and potentially masked group differences in subsequent palatable food consumption. Altogether, these findings suggest a complex pattern of sex- and age-specific effects on sucrose and sweet palatable food consumption following early life scarcity-adversity.

We found a sex-specific impact of early life scarcity-adversity on adolescent social play behavior. Specifically, adolescent males exposed to early life scarcity-adversity made fewer dorsal contacts compared to control males. Given that dorsal contacts are typically interpreted as a signal that the rat wants to initiate a play bout (Vanderschuren et al., 2016), a reduction in the number of dorsal contacts likely reflects a reduced motivation in scarcity-adversity males to engage in socially-rewarding interactions. These findings are in accordance with previous studies showing reductions in total social play levels in adolescent males following LBN exposure (Molet et al., 2016; Bolton et al., 2018a) and reduced adolescent social approach, as indexed by less time spent in a chamber containing a social stimulus animal, following scarcity-adversity (Raineki et al., 2015; Yan et al., 2017). Moreover, we extended these findings by showing no impact of early life scarcity-adversity in adolescent females, suggesting a sex-specific impact of early life scarcity-adversity on social behavior. In fact, this pattern of male-specific social deficits following ELA is further supported by recent work showing social preference deficits in adolescent male mice but not female mice raised in a resource-scarce environment (Maulik et al., 2023).

In sum, our results support a model where early life scarcity-adversity creates developmental vulnerabilities that unfold across distinct timeframes. The absence of peripubertal changes followed by sex- and reward-specific adolescent alterations suggests that early life scarcity-adversity may program vulnerabilities that require additional developmental triggers to manifest behaviorally. The sex-specific nature of our findings points towards several potential mechanistic pathways. The female vulnerability to consummatory reward deficits during adolescence may reflect interactions between ELA-induced stress system alterations and pubertal hormonal changes, particularly given the evidence that estrogen modulates dopaminergic reward processing (Yoest et al., 2014). The male-specific reduction in social play initiation may reflect ELA effects on developing social reward circuits or stress-sensitive regions like the prefrontal cortex, which undergoes prolonged maturation and regulates social behavior through downstream actions on neural circuits (Packard and Opendak, 2022; Ferrara et al., 2023; Mack et al., 2024).

### 4.3 Methodological considerations and limitations

While our study found that females show adolescent vulnerability to early life scarcity-adversity effects on sucrose preference, previous research has shown that adolescent males can also exhibit reduced sucrose preference following early life resource scarcity (Molet et al., 2016; Bolton et al., 2018a), although this was not observed in the current study. Discrepant findings across these studies and the current study may highlight the sensitivity of early life resource scarcity effects to experimental parameters including breeding and housing conditions, testing protocols, and the specific nature of the early life scarcity adversity paradigm and its effects on maternal behavior. For example, an interesting possibility is that the scarcity-adversity and LBN paradigms exert subtle differences in maternal behavior, with scarcity-adversity increasing adverse pup-directed maternal behavior and LBN increasing unpredictability of maternal patterns. These subtle differences in early experience may engage distinct stress response and neural systems and contribute to the varying outcomes across studies.

## 5 Conclusion

Our findings demonstrate that early life scarcity-adversity differentially affects reward-related consummatory and social behaviors depending on developmental timing, sex, and reward type. These age- and sex-specific effects advance our understanding of how early life experiences shape reward system development and identify critical developmental windows and sex-specific vulnerabilities that warrant further investigation.

## Data Availability

The original contributions presented in the study are included in the article/supplementary material, further inquiries can be directed to the corresponding author.
